# Estrogens and Their Receptors in Prostate Cancer: Therapeutic Implications

**DOI:** 10.3389/fonc.2018.00002

**Published:** 2018-01-18

**Authors:** Erika Di Zazzo, Giovanni Galasso, Pia Giovannelli, Marzia Di Donato, Gabriella Castoria

**Affiliations:** ^1^Department of Biochemistry, Biophysics and General Pathology, University of Campania Luigi Vanvitelli, Naples, Italy

**Keywords:** prostate cancer, castrate resistant prostate cancer, estradiol, estradiol receptors, new drugs

## Abstract

A major challenge in clinical management of prostate cancer (PC) is to limit tumor growth and prevent metastatic spreading. Considerable efforts have been made to discover new compounds for PC therapy and recent years have seen promising progress in this field. Pharmacological approaches have been designed to achieve benefits in PC treatment and avoid the negative side effects resulting from administration of antagonists or agonists or new drugs. Nonetheless, the currently available therapies frequently induce resistance and PC progresses toward castration-resistant forms that can be caused by the androgen receptor reactivation and/or mutations, or derangement of signaling pathways. Preclinical and clinical findings have also shown that other nuclear receptors are frequently altered in PC. In this review, we focus on the role of estradiol/estradiol receptor (ER) axis, which controls PC growth and progression. Selective targeting of ER subtypes (α or β) may be an attractive way to limit the growth and spreading of prostatic cancer cells.

## Introduction

Prostate cancer (PC) is one of the most frequently diagnosed cancers and a leading cause of cancer-related deaths in Europe, with around 417,000 new cases diagnosed in 2012. It also represents the second most common cancer worldwide for males, and the fourth most common cancer overall, with more than 1.1 million new cases diagnosed per year (World Cancer Research Fund International, http://www.wcrf.org).

Prostate cancer develops mainly in men aged 65 or older, and is rare before age 40. It is commonly considered a “hormone-dependent cancer,” since sex steroid hormones control PC initiation and progression. Preclinical and clinical findings have, indeed, highlighted the importance of circulating steroid levels in PC pathogenesis (1). PC is initially an androgen-dependent disease. At this early stage, androgen deprivation therapy (ADT) represents the major therapeutic option (2). Although initially effective in blocking tumor growth, this approach frequently fails, and the disease progresses to an androgen-independent state, also known as castration-resistant PC (CRPC), commonly characterized by poor prognosis and an average survival ranging from 10 to 20 months.

Castration-resistant PC can be caused by the reactivation of androgen receptor (AR) transcriptional activity due to AR gene amplification, or AR mutations, which lead to promiscuous binding of antiandrogens or other steroids for AR activation, or expression of different constitutively active AR splice variants. Derangements of signaling effectors (e.g., receptor and non-receptor tyrosine kinases) or scaffolds were also reported in CRPC (3).

Whatever the mechanism, few therapeutic targets have been identified in CRPC, and a limited number of therapies are, therefore, available. New efforts are needed to unravel the complex mechanism underlying PC hormone resistance and spreading.

Estrogens play an important role in male sex hormone secretion as well as in the growth, differentiation, and homeostasis of normal prostate tissues. They are also involved in prostate carcinogenesis (4, 5).

In this manuscript, we will review recent data on the role of estradiol/estradiol receptor (ER) axis in PC. These findings have opened the way for novel therapeutic strategies based on the use of ER agonists/antagonists and new compounds, whose efficacy has been successfully tested in preclinical and clinical models of PC.

## Estrogen Receptors: Structure and Function

Estrogen effects are commonly mediated by two receptors, estrogen receptor α (ERα) or β (ERβ), encoded by two separate genes [*ESR1* and *ESR2* (6)]. ERα and ERβ belong to the family of ligand-modulated transcription factors (TCFs), also known as nuclear receptors, often found altered in PC (7). As with other NR family members, ER proteins consist of an N-terminal ligand-independent transactivation domain (AF1; NTD), a DNA-binding domain (DBD), and a C-terminal ligand-binding domain (LBD) containing the ligand-dependent AF2 transactivation domain (8). ERα and ERβ share high sequence homology, particularly in the DBD, allowing both receptors to recognize the estrogen-responsive element (ERE) on DNA (9). In contrast, the LBD shows a lower sequence homology (58%) than the DBD, suggesting that ERα and ERβ have different specific ligands. ERβ shows a lower affinity for estradiol than ERα, while it exhibits a higher affinity for 4-hydroxytamoxifen, genistein, and the testosterone derivative 3β-androstanediol. In humans, there are at least five ERβ isoforms (ERβ1, 2, 3, 4, 5) generated by alternative splicing of exons 7 and 8 coding for the LBD and transactivation domain 2. Specifically, ERβ2 and ERβ5 proteins have truncated C-terminal regions, resulting in the loss of AF2 domains, and display differences in LBDs (10, 11). Among the isoforms of ERβ, only ERβ1 is functional, while the others control its activity. ERβ activity may, therefore, depend on ERβ1 expression and the ERβ isoform ratio.

Once activated by their ligands, ERs (α or β) mainly act through two types of signaling mechanisms: a classical, nuclear, or genomic mechanism and an extranuclear, non-genomic pathway. In the classical or genomic mechanism, estrogens diffuse across cell membranes and bind to their intranuclear and/or cytoplasmic receptor, which undergoes dimerization. The receptor(s) thus bind ERE sequences in the promoter region of target genes involved in cell proliferation, differentiation, and metabolism (12). In contrast, extranuclear/cytoplasmic receptors activate a rapid, non-transcriptional or non-genomic pathway upon estrogen binding. Szego and Davis observed, for the first time, a rapid increase in uterine cAMP within 15 s after treatment with physiological doses of estradiol (13). Data collected over the last decade show that extranuclear ERs trigger the rapid activation of various signaling pathways, causing different hormonal effects upon ligand binding (14). We now know, however, that there is a co-operation between genomic and non-genomic pathways (15). Thus, non-transcriptional routes control transcriptional routes and vice versa. The balance and integration between the different mechanisms (transcriptional versus non transcriptional) might play a role in pathophysiological processes, such as proliferative diseases of breast and prostate tissues, inflammatory and immune response, wound healing, cardiovascular and neurodegenerative disease, osteoporosis, and cellular aging (16).

## Estrogens in Prostate Carcinogenesis

Preclinical findings have shown that estradiol levels play an important role in PC pathogenesis. In aromatase knockout (KO) mice, which cannot metabolize androgens to estrogens, high testosterone levels only lead to prostatic hypertrophy and hyperplasia. In contrast, high estrogen and low testosterone levels induce inflammatory events and premalignant lesions (17). These findings are corroborated by epidemiological studies, suggesting that estradiol serum levels and estradiol/testosterone (E/T) serum ratio impinge on PC initiation and progression. African-American men, who have high serum estradiol levels, exhibit a greater risk of developing PC (18), and PC incidence increases during aging, since it is often diagnosed in elderly rather than young men (19). In elderly males, testosterone production by the testis declines, while estradiol concentration remains constant (20). Consequently, the ratio between circulating and intraprostatic E/T increases.

Different mechanisms have been proposed to explain the change in E/T ratio. In PC, *in situ* production of estrogen increases (21) and prostatic aromatase can be aberrantly expressed (22). Again, aromatization of androgens to estrogens in adipose tissue may also account for the modification in E/T ratio (23). This hypothesis is supported by the increase in female-type fat observed in elderly males. Unlike the aromatase promoter in the gonads, aromatase expression is regulated by cytokines and tumor necrosis factor (TNF) α in adipose tissue (24). Indeed, estrogen levels increase in men during inflammatory processes and obesity (25). This hormonal fluctuation might explain the relationship between inflammation, obesity, and PC frequently observed in elderly men. Altogether, these findings suggest that prostate tissue is exposed to fluctuations in sex steroid (e.g., estrogens and androgens) levels. Thus, a decrease in androgen levels might cause prostate tissue atrophy, while androgen restoration might foster the regrowth of prostatic epithelium. In this way, the clonal expansion of cells harboring oncogenic mutations is enabled.

The aberrant expression of 5α-reductase (5α-R) enzymes also seems to play a role in PC development (26). These enzymes are expressed in prostate tissue, PC, and CPRC (27–29) and convert testosterone into the more active dihydrotestosterone (DHT) (30). As such, 5α-R enzymes might primarily control development of human benign or malignant prostatic diseases (30). The enzymes, however, also induce metabolization of DHT into 3β-androstanediol, the natural ligand of ERβ (31). Once activated by 3β-androstanediol, ERβ restrains epithelial growth by regulating prostate AR content (31) or upregulating the PUMA proapoptotic protein (32). In sum, the balance between DHT and 3β-androstenediol might foster or limit the growth of prostatic tissues.

From these and other findings in literature, it seems that the ligand levels and their fluctuations during adult life are likely a culprit in prostate tumorigenesis and PC progression.

## ERs in PC

Prostate tissue expresses both ERα and ERβ. ERα is mainly expressed in stromal cells within the non-malignant human prostate, though is occasionally found in basal-epithelial cells, whereas ERβ is mainly confined to basal-epithelial cells (33–35). Immunohistochemical studies revealed that PC specimens express both ER α and β, although their relative levels at different stages of PC still remain unknown (7).

Estradiol receptor α expression is significantly associated with high Gleason score and poor survival in PC patients (36), while the expression of ERβ was found decreased or lost in PC samples (37). Additionally, co-expression of ERβ2 and ERβ5, two isoforms of ERβ, represents a prognostic marker for biochemical relapse, postoperative metastasis, and time to metastasize after radical prostatectomy in PC patients (11). Consistent with these findings, ERβ1 expression decreases, while ERβ2 and ERβ5 expression increases as PC progresses. Such a change was correlated with PC spreading and metastasis (26).

Many investigations about the role of ER α or β in PC have been made and continue to emerge. Collectively, these findings suggest that ERα acts as an oncogene and mediates the harmful effects of estrogen, such as proliferation, inflammation, and prostate carcinogenesis. ERα KO mice do not develop high-grade prostate intraepithelial neoplasia or PC after testosterone and/or estrogen treatment (21). In contrast, ERβ, whose expression decreases by promoter DNA methylation as PC progresses, seems to play an anti-oncogenic role. Indeed, ERβ agonists prevent proliferation of prostatic epithelium (38). Again, Ricke and colleagues demonstrated the opposite role of ERs in prostate tissue transformation using as a model ERα or ERβ KO mice treated with androgen in combination with estrogen. While ERβ KO induced dysplastic changes and premalignant transformation, ERα KO mice remained PC-free (21). Other findings, however, support the protective role of ERβ in prostatic transformation. The loss of ERβ expression correlates, for instance, with increased proliferation in the ERβ KO/transgenic adenocarcinoma of mouse prostate mouse model (39) and human PC specimens (37, 40). Although ERβ is downregulated during PC progression (41), its upregulation or activation inhibits tumor progression and induces cell cycle arrest and apoptosis in PC (42). Estradiol treatment of ERβ-expressing LNCaP cells xenografted in mouse inhibits PC establishment and growth (43). ERβ activation also causes apoptosis in Gleason grade 7 xenografted tissues as well as in androgen-independent PC3 and DU145 cell lines (44). Altogether, the findings obtained to date in preclinical and clinical models suggest that ERβ manipulation by ligands or new drugs might be useful in PC therapeutic approaches, particularly during the initial stages of PC. No clonal expansion of cells harboring mutations occurs if the growth of early grade PC is restrained. Thus, prostatic cancerous cells have less chance of spreading and progressing to metastatic phenotype.

Despite these findings, ERβ seems to foster cell cycle progression and DNA synthesis of PC cells. A rapid estradiol action mediated by the assembly of ERβ/AR/Src ternary complex drives cyclin D1 upregulation, cell cycle progression, and proliferation of PC-derived LNCaP cells (45, 46). Notably, extranuclear action mediated by ERβ increases the mitogenesis and motility of LNCaP cells challenged with epidermal growth factor (EGF), suggesting that ERβ also intersects EGF and its cognate receptor in PC cells (47). This finding is important, since CRPC often exhibits derangements of EGF-receptor signaling (48). Again, estradiol and the ERβ-selective agonist diarylpropionitrile both increase DNA synthesis and cyclin D2 expression in androgen-independent PC3 cells, suggesting that ERβ mediates the estrogen proliferative effect in these cells. As well as being blocked by the ERβ-selective antagonist 4-[2-Phenyl-5,7-bis(trifluoromethyl) pyrazolo[1,5-a]pyrimidin-3-yl]phenol (PHTPP), these effects are also inhibited by PKF118-310, a compound that disrupts β-catenin/T-cell-specific TCF complex, suggesting the involvement of β-catenin in the effect mediated by estradiol/ERβ axis in PC3 cells (49, 50).

**Figure 1:**
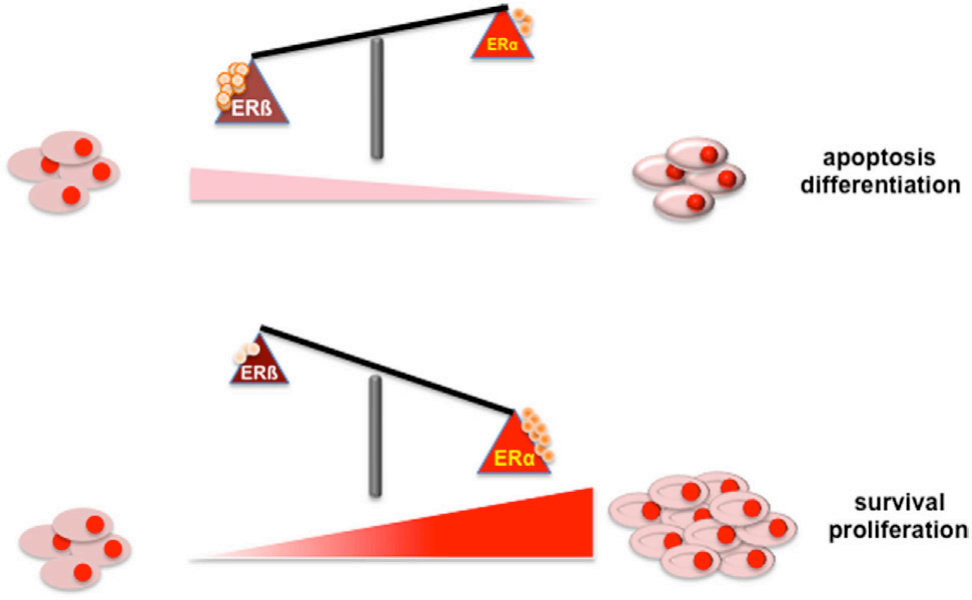
Illustrates the putative role of ER (α or β) in PC.

Figure 1 depicts the putative role of ER (α or β) in PC. Findings here discussed indicate that apoptosis and/or differentiation of PC cells might be promoted when ERβ increases or is activated (upper section). In contrast, survival and proliferation of PC cell might be enhanced by the increase in ERα content or its activation (lower section).

The conflicting results described above on the role of ERs (α or β) in PC pathogenesis can be reconciled if the observed biological effects are dependent on the experimental model used. A major consideration is how closely PC models currently used (e.g., genetically engineered mouse, xenograft, and cell culture models) recapitulate important features of human prostate and human PC. Mouse and human prostate gland are, in fact, quite different in structure (51). Again, the lack of PC cell models able to faithfully reproduce the complex crosstalk between AR, ERα, ERβ, and EGF-R described at non-transcriptional level in primary and PC-derived cells (45–47, 52) could also explain the discrepancies observed in cultured cells.

**Figure 2:**
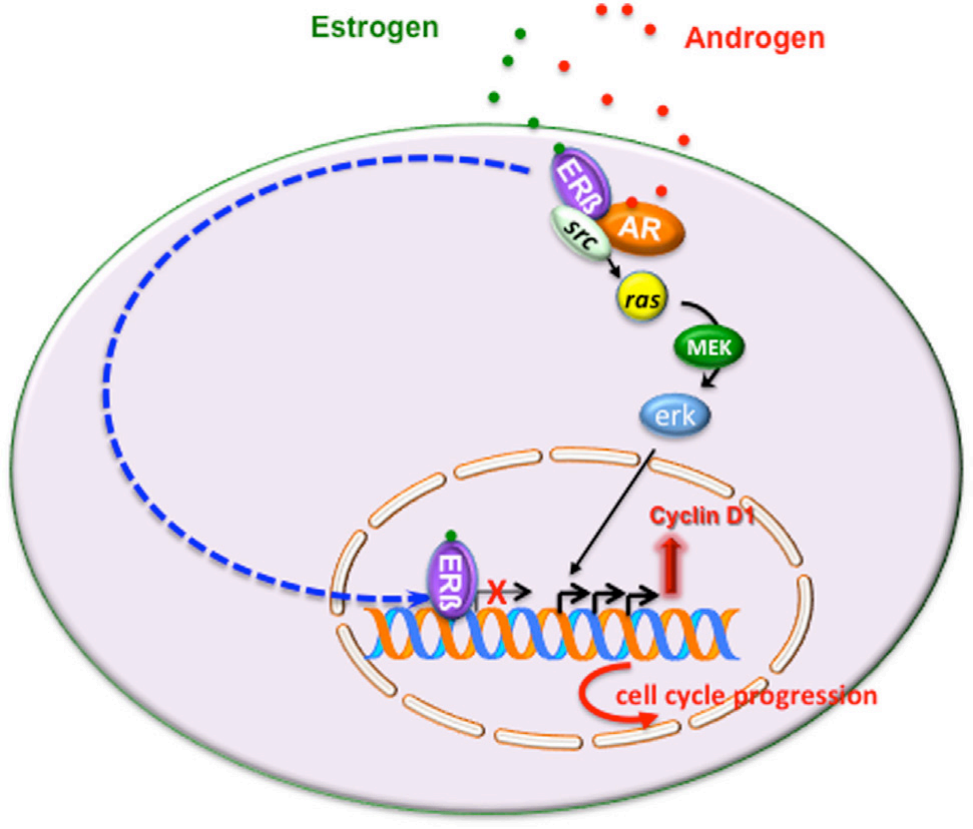
Estrogen or androgen stimulation rapidly leads to extranuclear assembly of estrogen receptor (ERβ)/androgen receptor (AR)/Src ternary complex in LNCaP cells. Once fully activated, Src tyrosine kinase triggers activation of Ras and its dependent kinase cascade. Activation of this signaling pathway increases cyclin D1 expression and promotes cell cycle progression in LNCaP cells. As discussed in the main text, the transcriptional activity mediated by ERβ might overcome the non-transcriptional activity of the receptor, thereby leading to cell cycle arrest and/or apoptosis.

Figure 2 illustrates the intricate extranuclear network activated by ERβ/AR/Src complex and leading to cyclin D1 increase and cell cycle progression of LNCaP cells challenged with estradiol or androgens.

Noteworthy, some PC cells are unable to replicate the transcriptional interplay between ERα/ERβ (53, 54), and the most routinely used PC cells do not represent prostatic tissue, since in human prostate ERα expression is confined to stromal cells, while ERβ is expressed in the luminal and basal epithelial cells. Additionally, the presence of ERβ inhibitors or the altered expression of transcriptional regulators (co-activators or co-repressors) in PC can also explain the conflicting findings observed to date.

In sum, it might be argued that under certain conditions, which depend on ERβ content or the ERα/ERβ ratio or ligand concentration or the co-regulator availability, the transcriptional mechanism mediated by ERβ overcomes the non-transcriptional mechanism, thereby leading to cell cycle arrest or apoptosis (Figure 2).

Although still debated, collected findings are of potential clinical interest because of the paucity of targets and therapeutics in advanced PC.

## Targeting ERs in PC: Therapeutic Approaches

Androgen deprivation therapy, either medical or surgical, is the first line of treatment for PC. Although more potent AR antagonists have been designed, resistance to these drugs continues to develop, resulting in CRPC, which is almost untreatable (55). Alternative approaches to androgen ablation need to be considered to prevent progression and metastatic spreading of PC.

Selective ER modulators (SERMs) are synthetic estrogen receptor ligands that show both estrogenic and/or antiestrogenic effects, depending on cell type and the different expression and/or activation of transcriptional co-regulators. By inducing a conformational change in ERs, SERMs might influence the interaction between ER and co-regulatory proteins [either co-activators or co-repressors (56)]. Several SERMS, such as tamoxifen, toremifene, and raloxifene, are able to suppress PC growth in mouse models and cultured cells (57–61). However, clinical trials with high-dose tamoxifen or toremifene did not show significant effects (62–64), and the combinatorial use of toremifene and ADT in patients with advanced PC has only recently been found to have a relative beneficial effect (65). Further, clinical trials with larger cohorts are needed to confirm these promising phase IIA results.

Raloxifene exerts estrogen agonist/antagonist effects by binding both ERα and ERβ. It induces the selective activation of ERβ and consequently apoptosis in both androgen-dependent (LNCaP) and -independent [PC3 and DU145 (58, 59)] cell lines. SERMs lower Bcl-2 expression, increase caspase-3 and Par-4 levels (66), and antagonize ERα, causing growth inhibition of human xenografted CWR22 and CWRSA9 PC cell lines (60, 67). However, the molecular events involved in the control of prostate carcinogenesis by raloxifene are still unclear. ICI 182,780, another member of the SERM family, exerts a dose-dependent growth inhibition effect on DU145 cells, which is mediated by the binding of ERβ to NF-κB and enhancement of TCF FOXO1 (68).

Altogether, the findings here discussed reveal many inconsistencies between *in vivo* and *in vitro* studies. It can be argued that pharmacokinetic differences in SERMs between humans and mice, which are often used as a study model, already exist. Again, therapeutic strategies may prevent PC progression at early stages of development, but might exert different or even opposite effects in CRPC. Conflicting data might also result from the differential expression profiles of AR, ERα, and ERβ shown by PC cell lines (e.g., LNCaP, PC3, and DU145) commonly used for *in vitro* studies. However, other additional factors (e.g., cell line cross-contamination, absence of cell line authentication, number of cell line passages in culture, contamination with microorganisms, genetic and phenotypic instability) might significantly influence the experimental results (69). Additionally, the cell culture media used for *in vitro* studies should be free from substances that compete for the sex steroid receptor binding activity and that may lead to false (both positive and negative) results. Furthermore, PC-derived cell lines do not faithfully reproduce human PC and are unable to recapitulate events caused by the presence of tumor stroma and/or extracellular matrix (70). Consistent with this hypothesis, depletion of a p53 mutant phenotypically reverts breast cancer cells in three-dimensional (3D), but not in bidimensional (2D) model (71). Thus 3D models, made up of epithelial/stromal cells and extracellular matrix, might help to unmask any weaknesses or potential resistance mechanisms of PC, which would otherwise only be detected in late drug discovery stage *in vivo*. Furthermore, studies in patient-derived xenografts, an approach that is currently emerging to discover new biomarkers and drugs in human cancers (72), might help to exactly recapitulate the PC biology in patients.

As discussed above, ERβ can be considered a tumor suppressor, and use of its agonist may, therefore, be effective in therapeutic approaches against PC. By using AR knockout mice to investigate the therapeutic effect of 8β-VE2, a potent synthetic selective agonist for ERβ, ERβ was found to be responsible for androgen-independent apoptosis in prostate stroma and epithelia, an effect that requires TNFα signaling. Moreover, the same study showed that 8β-VE2-activated ERβ induces apoptosis in androgen-independent PC3 and DU145 cells, as well as in primary human PC xenografts (44).

Treatment with the ERβ agonist 8β-VE2 induces beneficial effects over current AR-targeting therapies by decreasing survival of CRPC cells and inhibiting the biological effects mediated by AR variants in the VCaP model of PC bone metastasis (50). Additionally, using the ERβ-selective agonist LY3201, it was shown that ERβ downregulates AR signaling, while upregulating the tumor suppressor PTEN (73).

In sum, collected findings support the idea that ERβ counteracts AR-mediated signaling, leading to cell proliferation and an inflammatory environment. Further, they strongly suggest that ERβ-selective agonists may be used to prevent fibrosis as well as development of benign prostatic hyperplasia, and to treat PC progression.

## Concluding Remarks

A major challenge in clinical management of PC is to limit tumor progression. ERs are differently distributed and exert different functions in the body. Targeting of different ER subtypes may provide a way to progress toward a more precise and tailored approach to hormone-related proliferative diseases, which depend, at least in part, on ERs. Unfortunately, available PC models are unable to fully reproduce molecular events underlying PC development and progression. While the AR status of the most commonly used cell lines is generally known, expression levels of ERα and ERβ vary across different cell lines, and conflicting data have been reported for each cell type, mainly as a result of the limitations of currently used approaches. The balance between the activity of AR and ERs, and their interaction, constitutes a regulatory network that enhances or limits the efficacy of drugs targeting steroid receptors.

A number of findings in literature support the idea that ERβ agonists may be beneficial at early stages of PC by keeping tumors to a low grade. A wide range of ERβ-selective agonists is currently available (74). They appear safe and do not affect proliferation in breast or uterus, while they do inhibit proliferation of prostate epithelia, without side effects on the pituitary (6). Additional research is still expected in the next years.

Understanding the factors that control ER functions in various PC cell types or in different mouse models and the crosstalk between ERs and their upstream or downstream partners represents an exciting prospect for treating prostatic diseases. Analysis of these interactions could lead to specific targeting of ER functions in living cells and patients. New drugs synthesized in different labs (46, 75–81) selectively target the interactions between steroid receptors and signaling effectors or scaffolds or transcriptional regulators. Further investigation into ER-interacting partners should identify other promising candidates to track and target in PC.

## Author Contributions

Conceptualization, supervision, and funding acquisition: GC. Writing—original draft: EZ and GG. Writing—review and editing: EZ, GG, PG, and MD.

## Conflict of Interest Statement

The authors declare that the research was conducted in the absence of any commercial or financial relationships that could be construed as a potential conflict of interest.
